# Changes of Plasma Amino Acid Profiles in Infants With a Nutrient-Fortified Complementary Food Supplement: Evidence From a 12-Month Single-Blind Cluster-Randomized Controlled Trial

**DOI:** 10.3389/fnut.2021.606002

**Published:** 2021-09-30

**Authors:** Chie Furuta, Wataru Sato, Hitoshi Murakami, Devika J. Suri, Gloria E. Otoo, Kwaku Tano-Debrah, Shibani A. Ghosh

**Affiliations:** ^1^Institute of Food Science and Technologies, Ajinomoto Co. Inc., Kawasaki-city, Japan; ^2^Research Institute for Bioscience Products and Fine Chemicals, Ajinomoto Co. Inc., Kawasaki-city, Japan; ^3^Nevin Scrimshaw International Nutrition Foundation, Boston, MA, United States; ^4^Department of Nutrition and Food Science, University of Ghana, Legon-Accra, Ghana; ^5^Friedman School of Nutrition Science and Policy, Tufts University, Boston, MA, United States

**Keywords:** complementary food supplement, stunting, plasma essential amino acids, plasma branched-chain amino acids, length-for-age z-score

## Abstract

Stunting is reportedly associated with low circulating levels of essential amino acids (EAAs). This study examined the effect of a macronutrient- and micronutrient-fortified complementary food supplement (KOKO Plus) on specific plasma EAA levels and stunting in infants aged 6–18 months. In a single-blind cluster-randomized controlled trial conducted in Ghana, infants were enrolled at 6 months and followed until 18 months. Thirty-eight communities were randomly assigned to receive KOKO Plus (KP, fourteen communities, *n* = 321), multiple-micronutrient powder (MN, thirteen communities, *n* = 327), or only nutritional education as control group (NE, eleven communities, *n* = 318), and all groups received nutrition education. Plasma amino acids (AAs) were measured at 6, 12, and 18 months (end point). Mixed-effects models were used to assess the effect of the intervention on plasma AAs, and the relationship between plasma branched-chain AAs (BCAAs) and the risk of stunting was assessed. At the end point, total BCAA concentrations (±standard error) significantly exceeded baseline in the KP (284.2 ± 4.3 μM) and NE (289.1 ± 4.4 μM) groups but not the MN group (264.4 ± 4.1 μM). After adjustment for compliance at 200 sachets, plasma BCAAs exceeded in the KP group (284.5 ± 4.2 μM) compared to the MN group (264.6 ± 4 μM). Plasma BCAAs were positively correlated with changes in length-for-age Z-score from baseline (*R* = 0.327, *p* = 0.048). In conclusion, the plasma BCAA concentrations of infants that received KP and the NE group was significantly higher compared to the MN group but there were no differences between the KP and NE group at end point. Improved plasma BCAAs may be due to improved nutrient intake by infants exposed to KP or NE. Low BCAAs were associated with stunting, replicating the previous finding.

**Clinical Trial Registration:** https://clinicaltrials.gov/ct2/show/NCT03181178?term=NCT03181178&draw=2&rank=1, identifier: NCT03181178.

## Introduction

Malnutrition is a global nutrition issue; a total of 155 million children under age 5 suffer from stunting due to malnutrition (1, 2). Once children are stunted, recovery becomes difficult, resulting in delays of growth and development, reduced cognitive function, decreased productivity during adulthood, and other consequences (3). The length-for-age Z-score (LAZ); which is an indicator to measure stunting, of malnourished children drops early after birth and continues to fall until 24 months of age (4); thus, exclusive breastfeeding practices and the quality and quantity of introduced complementary foods become critical. Recent literature points to the importance of the quality of protein in complementary foods (5) and its correlation with stunting. Analysis of Malawian children has found a strong negative correlation between all essential amino acids (EAAs), including branched-chain amino acids (BCAAs), and stunting (6). At the same time, higher levels of protein can be a trigger for increased insulin production and growth, which can be detrimental in the long term by increasing the risk of adiposity (7–9). Nonetheless, protein intake in early life is positively associated with height and weight at 10 years of age (10, 11).

In Ghana, where malnutrition is observed in some regions, stunting remains a significant issue, occurring in ~28% of children aged 24–35 months (12, 13). Data on the feeding on infants and young children show that for breastfed children aged 6 to 35 months, cereals are the first foods introduced in the diet. While Ghanaian infants also consume diverse food groups, those foods are introduced much later than the recommended age for the start of a high-quality diet (i.e., 6–8 months) (12, 13). The source of protein in complementary foods and the consumption of breast milk vs. formula might also affect the relationship among protein intake, linear growth and adiposity (14). In a recent study from Ethiopia, intake levels of tryptophan, protein, and energy as well as serum levels of insulin growth factor-1 and transthyretin were positively associated with linear growth, implying a potential role for amino acids and high-quality protein in improving linear growth (15).

An analysis of the effect of a macronutrient- and micronutrient-fortified complementary food supplement called KOKO Plus (KP) on linear growth demonstrated a dose–response effect (16). Given the composition of KP as well as recent findings, assessing plasma amino acid levels and the effect of supplementation might elucidate information that would be critical for an improved understanding of the modifiable factors related to linear growth. Essential amino acids are needed for protein synthesis, in particular, branched-chain amino acids (leucine, isoleucine, and valine) play a critical role since leucine is primarily responsible for activating protein synthesis *via* the mammalian target of rapamycin signaling pathway (17). However, the BCAA balance is in fact important to enhance protein synthesis since leucine activates the metabolic pathways that oxidates all BCAAs (18). Thus, this study reports on a secondary analysis of the study conducted in Ghana to examine the effect of providing KP to children from 6 months to 18 months of age (a 12-month intervention period), coupled with nutrition education (NE) to targeting mothers, on plasma amino acids compared to the effects of a mutiple-micronutrient powder (MN) and maternal NE or maternal NE alone. We hypothesized that children who received KP would have higher plasma BCAA concentrations than children receiving MN plus NE and NE alone at end point. In addition, we sought to clarify the relationship between plasma EAAs, especially BCAAs, and LAZ in infants. We hypothesized that plasma EAA and BCAA levels would be positively correlated with LAZ in infants.

## Materials and Methods

### Trial Design and Interventions

#### Trial Design

The study was a secondary analysis using data from a cluster-randomized single-blind trial conducted in the Central Region of Ghana from 2013 to 2015 to examine the effect of a macronutrient and micronutrient supplement, KP, on growth, micronutrient status and morbidity. The study compared the KP intervention, which included provision of KP sachets for daily use and NE targeting mothers, with the provision of a MN and NE or the provision of NE alone. The 12-month intervention, targeting mothers and infants, began when the infants turned 6 months old and ended when they turned 18 months old. One group received KP with NE, a second group received MN with NE, and a third group which is the control group received NE alone (16).

#### Ethics

The study protocol was reviewed and approved by the Institutional Review Boards of the Ghana Health Service and the Noguchi Memorial Institute for Medical Research at the University of Ghana. A data safety monitoring board composed of faculty and academics from the University of Ghana (who were not involved in the research) monitored the progress of the trial. Written informed consent was received from each parent/caregiver. No interim analyses were planned, and no stopping rule was predefined. No serious side effects were detected, and no reason to interrupt the study was identified. This trial was registered on ClinicalTrials.gov as NCT03181178 on June 8, 2017. All experiments were also performed in accordance with relevant guidelines and regulations.

#### Interventions

The specified dose of KP contained 30% of the recommended daily energy, 60% of the total protein, 35–55% of the total amino acids and 40% of the total fat recommended by the WHO complementary feeding guidelines (19, 20). The micronutrient premix in both KP and micronutrient powder provided 50–150% of UNICEF's Recommended Nutrient Intakes (21). The ingredients and nutrient composition of KP and MN have been presented in prior publications (22, 23). The supplements were formulated for daily consumption and were distributed on a weekly basis. The nutrient education component consisted of monthly mothers and infants' sessions including role-plays, activities and cooking demonstrations by Ghana Health Service (GHS) volunteers. The educational materials were adapted from the Good Life project, a US Agency for International Development behavior change project conducted from 2009 to 2013, to support GHS in areas of family planning, maternal and child health, malaria, nutrition, water, and sanitation with some modifications so it can follow the trial (24). Further details of the trial design and interventions are described elsewhere (16).

### Participants, Setting, Sample Size, and Randomization

#### Participants

**Figure 1:**
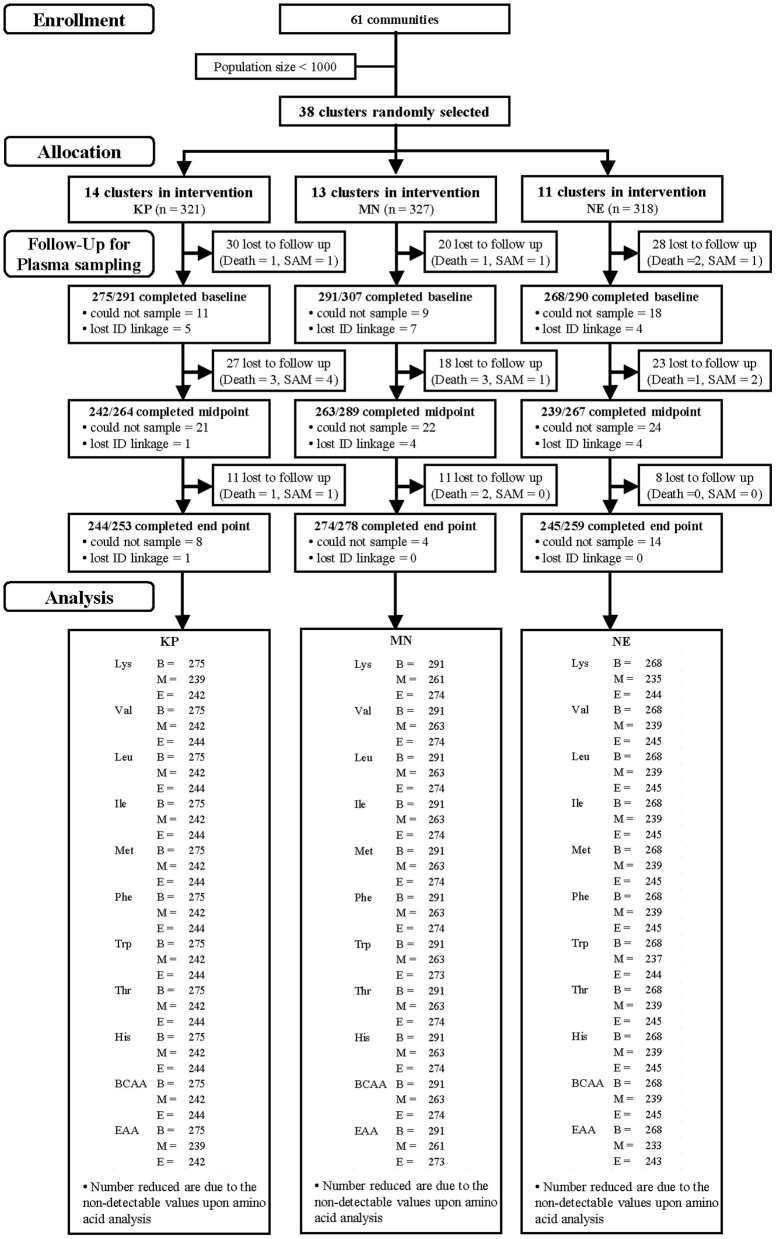
Study participants and follow-up by group [KOKO Plus (KP), micronutrient powder (MN) and nutrition education (NE)]. *Lost to follow-up includes deaths and severe acute malnutrition (SAM).

Participants from 39 communities in three districts of the Central Region of Ghana were recruited. The inclusion criteria were singleton term birth, breastfeeding as the exclusive or predominant nutrition source, parents' intention to live in the same community for a period of at least 12 months, and parents' willingness to participate for the entire study period. The recruitment of study participants started in February 2013, and follow-up was completed at the end of February 2015. Actual study flow, loss to follow-up and “drop outs” by individual participants, sample collection and sample analysis is shown in Figure 1.

#### Setting

Infant and young-child feeding data in Ghana shows cereals are predominantly the first foods introduced in the diet in breast-fed children ranging 6–35 months of age (12). A qualitative study was conducted to understand the situation, issues on complementary feeding, health, and food security, the Cape Coast region where the study was located and in the rest of the two regions studied (Greater Accra and Upper West), koko was the most preferred first food because of its affordability and ease of preparation (22, 25). Koko is a fermented maize/corn porridge which is well-consumed in Ghana and has been found to be poor in energy density (about 25 kcal/100 g) and protein quality, and low in micronutrients and has been implicated in protein-energy malnutrition (26).

#### Sample Size

For the secondary analysis, we calculated the sample size needed to detect a 5% change in plasma BCAAs among the treatment groups at 80% power. Calculations were conducted using the “TrialSize” (ver. 1.3) package for R (https://CRAN.R-project.org/package=TrialSize).

Using standard deviations (SDs) of plasma valine, leucine and isoleucine established in a previous study (27), the SD of plasma BCAAs was calculated as 32.7. Alpha was set at 0.05. In order to detect at least a 5% change [19 μM in the previous study (27)] in plasma BCAAs between groups, the required sample size was 63 per group. The design effect was 1.66, assuming 23 participants per cluster and an intra-cluster correlation coefficient of 0.03 with an attrition rate of 15%. These inputs led to a total sample size of 123 per group [*n* = 63 × 1.66 × (100/85)].

This study was embedded in a primary study that had a sample size of 301 per group (final sample size achieved= 298) (16), which exceeded the sample size required for our secondary analysis. We thus retained the original sample size for the analysis of plasma amino acids (a final sample size of *n* = 298).

#### Randomization

Among 61 communities in the Central Region of Ghana, 39 communities were selected by a research associate using the Microsoft Excel (Microsoft Excel, RRID:SCR_016137) random number function (RAND). Subsequently, a new random sequence was generated using RAND followed by block randomization, and the clusters were randomly assigned to three groups (KP, MN, and NE) by the same research associate. Changes occurred in the total number of clusters per group as study implementation began. Ultimately, the total number of clusters was 38, with 14 in the KP, 13 in the MN (original allocation) and 11 in the NE group (16).

### Sample Collection

Blood sampling for amino acid analysis was performed at each time point (when the subjects reached 6, 12, 18 months, baseline, midpoint, end point, respectively), on all subjects who participated in the main study. The length of time since the last meal was asked and recorded at blood sampling. A flow diagram representing the distribution of participants is shown in Figure 1. Trained pediatric phlebotomists collected venous blood for plasma, using K_2_EDTA as an anticoagulant. The samples were immediately placed in a CubeCooler (Forte Grow Medical Co. Ltd., Tochigi, Japan) to achieve a steady temperature of 4°C (28) and were transported back to the laboratory within 5 h of collection.

The K_2_EDTA blood was centrifuged at 3,000 *g* for 14 min at 4°C, and the plasma was aliquoted into Eppendorf tubes. The plasma was mixed with 2 volumes of 5% (w/w) trichloroacetic acid and centrifuged immediately (4°C, 20 min, 8,000 *g*) to remove the precipitated protein. The samples were stored at −80°C until they were shipped to Japan for the measurement of amino acid concentrations. During transport, the samples were kept frozen in cooler boxes with dry ice.

### Amino Acid Analysis

The concentrations of several amino acids (methionine, leucine, valine, isoleucine, lysine, phenylalanine, tryptophan, threonine, and histidine) were measured using an automatic amino acid analyser (L-8800; Hitachi High-Technologies Corporation., Tokyo, Japan). Amino acids were separated by cation-exchange chromatography on a cross-linked sulfonated polystyrene resin column with a modified lithium citrate buffer system (29). After separation, the amino acids were reacted with ninhydrin reagent and detected spectrophotometrically (absorption maximum 570 nm) as their derivatives (30). The analysis was started in September 2014 and completed in November 2015. The samples were analyzed separately in several batches in the order of the arrival to the lab.

Total BCAA (leucine, valine and isoleucine) and EAA (methionine, leucine, valine, isoleucine, lysine, phenylalanine, tryptophan, threonine, histidine and cystine) concentrations were calculated as the sum of the concentrations of all BCAAs or all EAAs. Plasma amino acid concentrations are given in micromolar (μM). Inter-assay and intra-assay coefficients of variation for each amino acid are shown in Supplementary Table 1.

### Data Analysis

Means with SDs or standard errors (SEs) or proportions with 95% confidence intervals (CIs) were used to describe the baseline, midpoint and end point parameters, as appropriate. Tukey's multiple comparisons test or Fisher's exact test was used to compare baseline characteristics.

Plasma amino acid analysis took into account the cluster-randomized design by using mixed models that recognized the multilevel structure of the data, where individual children were nested within the 39 communities. A linear mixed-effects model was used to predict plasma amino acid concentrations at each time point (31). We used the predicted value and its SE instead of the actual value as a value that appropriately excluded the cluster-dependent variation and bias. Fixed effects in the model included the treatment (KP, MN or NE), time points (baseline, midpoint or end point) and their interaction. The model was adjusted for baseline values and the random effects of clusters. The following generic model was run:


$$\begin{array}{l} \text{Y}=\beta_{0\left(\text{intercept}\right)}+\beta_{1\left(\text{treatment}\right)}\;+\;\beta_{2\left(\text{time point}\right)}\\ +\beta_{3\left(\text{treatment }\times \text{ time point}\right)}+\beta_{4\left(\text{baseline value}\right)}+r_{\left(\text{community}\right)}\\ {\text{r}}_{\left(\text{community}\right)}\sim N\left(0,\sigma^{2}\right) \end{array}$$

In order to determine the model coefficients and their covariance matrixes, the model was fitted by a residual maximum likelihood estimation with the “lme4” (ver. 1.1.13) R package [RRID:SCR_015654 (32)]. Estimated coefficients and covariance matrixes were used to predict amino acid concentrations. The predicted plasma amino acid concentrations were compared between each pair of treatment groups using the “multcomp” (ver. 1.4.6) R package [RRID:SCR_018255 (33)]. The *p*-value of comparison was adjusted by the Bonferroni method (the *p*-value multiply by a repeated number of statistical tests) and α = 0.05 was used for significance level.

Community means were used for the correlation analyses between delta LAZ and delta plasma BCAA concentration to handle the issue of statistical independence among individuals within a cluster. The Pearson correlation coefficient was calculated.

In this study, the participants' compliance rate in the use of supplements, that is, the reported consumption of supplements delivered, was 84.9% (KP) and 87.2% (MN), but the delivery rates were ~60%; therefore, the absolute number of sachets consumed was, on average, 186 out of the target 365 sachets (51%) over the one-year study period (16). Given the insufficient delivery rate of the supplements, we assessed the effect of supplement consumption on plasma amino acid concentrations using a generic model (specified below):


$$\begin{array}{l} Y=\beta_{0\left(\text{intercept}\right)}+\beta_{1\left(\text{treatment}\right)}+\beta_{2\left(\text{time point}\right)}+\beta_{3\left(\text{supplements consumed}\right)}\\ +\beta_{4\left(\text{treatment }\times \text{ time point}\right)}+\beta_{5\left(\text{treatment }\times \text{ supplements consumed}\right)}\\ +\beta_{6\left(\text{time point }\times \text{ supplements consumed}\right)}\\ +\beta_{7\left(\text{treatment }\times \text{ time point }\times \text{ supplements consumed}\right)}+{\text{r}}_{\left(\text{community}\right)}\\ {\text{r}}_{\left(\text{community}\right)}\sim N\left(0,\sigma^{2}\right) \end{array}$$

Fixed effects in the model included the treatment (KP, MN or NE), time points (baseline, midpoint or end point), supplements consumed and their interaction. The model was adjusted for baseline values and the random effects of clusters. The model coefficients and their covariance matrices were calculated in the same way as above. The calculated parameters were then used to estimate plasma amino acid concentrations assuming different consumption levels by each infant (for example, 100, 200, or 300 sachets). The predicted plasma amino acid concentrations were compared as above. All statistical analyses were performed using the R (ver. 3.3.0) platform (RRID:SCR_001905, https://www.r-project.org/).

## Results

### Baseline Characteristics

Table 1 presents the baseline characteristics of the infants included in the analyses. There were no significant differences (*p* > 0.05) between groups in any of the characteristics at time point baseline.

**Table 1:** Baseline characteristics for children included in the plasma amino acids analysis^a^.

**variables**	**KP**	**MN**	**NE**
Participants, n	275	291	268
Male sex, n	139 (50.5%)	139 (47.8%)	136 (50.7%)
Age, mo	6.1 ± 0.6	6.2 ± 0.5	6.2 ± 0.7
Height, cm	65.1 ± 2.7	65.2 ± 2.5	65.5 ± 2.6
Weight, kg	7.1 ± 1.0	7.1 ± 1.0	7.2 ± 1.0
MUAC, cm	14.0 ± 1.2	14.0 ± 1.1	14.1 ± 1.1
Height-for-age z score	−0.76 ± 1.03	−0.74 ± 0.96	−0.65 ± 1.08
Weight-for-age z score	−0.72 ± 1.16	−0.69 ± 1.09	−0.67 ± 1.11

a *Values are expressed as n (%) or means ± SDs unless otherwise indicated. There are no significant difference between all groups. KP, KOKO Plus; MN, Micronutrient; NE, Nutrition Education; MUAC, Mid-upper arm circumference*.

### Plasma Amino Acid Concentrations

Summary of the raw data on plasma amino acids are shown in Supplementary Table 2. Table 2 presents the predicted plasma amino acid concentrations at baseline, midpoint and end point. At baseline, there was no significant difference (*p* > 0.05) in the concentration of any of the EAAs among the 3 groups.

**Table 2:** Predicted plasma amino acids concentration in each time point.

	**Baseline**						**Midpoint**						**End point**					
	**Predicted plasma concentration^a^**			***P*** **^b^**			**Predicted plasma concentration^a^**			***P*** **^b^**			**Predicted plasma concentration^a^**			***P*** **^b^**		
**Amino acids**	**KP**	**MN**	**NE**	**KP vs. MN**	**KP vs. NE**	**MN vs. NE**	**KP**	**MN**	**NE**	**KP vs. MN**	**KP vs. NE**	**MN vs. NE**	**KP**	**MN**	**NE**	**KP vs. MN**	**KP vs. NE**	**MN vs. NE**
Lysine	119.5 ± 2.0	121.4 ± 2.0	120.7 ± 2.1	1	1	1	121.5 ± 2.2	111.8 ± 2.1	114.4 ± 2.3	**0.004**	0.072	1	111.8 ± 2.2	107.1 ± 2.1	107.8 ± 2.2	0.36	0.61	1
Valine	133.0 ± 2.1	134.5 ± 2.1	135.8 ± 2.2	1	1	1	145.8 ± 2.2	134.5 ± 2.2	142.8 ± 2.3	**<0.001**	1	**0.026**	150.0 ± 2.2	141.5 ± 2.2	152.4 ± 2.3	**0.017**	1	**0.002**
Leucine	85.9 ± 1.4	87.2 ± 1.4	87.8 ± 1.5	1	1	1	88.4 ± 1.5	79.3 ± 1.5	87.3 ± 1.6	**<0.001**	1	**<0.001**	86.6 ± 1.5	79.9 ± 1.5	89.4 ± 1.6	**0.005**	0.62	**<0.001**
Isoleucine	46.5 ± 0.8	47.4 ± 0.7	47.8 ± 0.8	1	0.72	1	49.4 ± 0.8	43.5 ± 0.8	47.2 ± 0.8	**<0.001**	0.18	**0.004**	47.6 ± 0.8	43.0 ± 0.8	47.2 ± 0.8	**<0.001**	1	**<0.001**
Phenylalanine	36.9 ± 0.7	37.2 ± 0.7	37.5 ± 0.8	1	1	1	45.7 ± 0.8	43.6 ± 0.8	45.0 ± 0.8	0.16	1	0.67	48.5 ± 0.8	45.6 ± 0.7	50.4 ± 0.8	**0.019**	0.27	**<0.001**
Tryptophan	34.0 ± 0.8	35.3 ± 0.7	34.2 ± 0.8	0.60	1	0.88	40.4 ± 0.8	36.9 ± 0.8	37.5 ± 0.8	**0.006**	**0.039**	1	29.3 ± 0.8	27.5 ± 0.8	30.3 ± 0.8	0.31	1	**0.035**
Threonine	100.7 ± 1.8	99.9 ± 1.8	101.4 ± 1.9	1	1	1	87.1 ± 2.0	77.9 ± 1.9	85.1 ± 2.0	**0.002**	1	**0.032**	77.6 ± 2.0	74.0 ± 1.9	77.1 ± 2.0	0.55	1	0.78
Histidine	59.2 ± 0.8	60.1 ± 0.8	59.7 ± 0.8	1	1	1	66.7 ± 0.9	61.7 ± 0.8	64.3 ± 0.9	**<0.001**	0.15	0.11	63.7 ± 0.9	62.3 ± 0.8	64.8 ± 0.9	0.66	1	0.097
Methionine	17.6 ± 0.3	17.8 ± 0.3	17.5 ± 0.3	1	1	1	18.4 ± 0.3	17.0 ± 0.3	18.1 ± 0.3	**0.004**	1	**0.037**	17.0 ± 0.3	16.6 ± 0.3	17.3 ± 0.3	0.99	1	0.29
BCAA	265.5 ± 4.0	269.2 ± 3.9	271.5 ± 4.1	1	0.89	1	283.7 ± 4.3	257.2 ± 4.2	277.4 ± 4.4	**<0.001**	0.92	**0.003**	284.2 ± 4.3	264.4 ± 4.1	289.1 ± 4.4	**0.003**	1	**<0.001**
EAA	633.5 ± 8.1	641.0 ± 7.9	642.7 ± 8.3	1	1	1	664.3 ± 8.8	606.6 ± 8.5	643.3 ± 9.1	**<0.001**	0.29	**0.009**	631.3 ± 8.8	598.6 ± 8.3	639.0 ± 8.9	**0.021**	1	**0.003**

a *Plasma amino acid concentrations were predicted from mixed model with a random intercept and random slope. Fixed effects were treatment (KP, MN, NE) and time points (baseline, midpoint, end point). The model was adjusted for baseline value and random effect of cluster communities. Predicted concentrations were expressed as means ± SEs [μM]*.

b *P-values were obtained from general linear hypotheses test (null hypothesis: difference between groups = 0) with the Bonferroni correction. Bold text indicates a statistically significant difference between groups with a p < 0.05. B, Baseline; M, Midpoint; E, End point; KP, KOKO Plus; MN, Micronutrient; NE, Nutrition Education; BCAA, Branch chain amino acid; EAA, Essential amino acid*.

At midpoint, the plasma lysine concentration (± SE) of the KP group (121.5 ± 2.2 μM) were significant higher values compared to that of the MN group (111.8 ± 2.1 μM) and higher in a trend-level compared to the NE group (114.4 ± 2.3 μM). At end point, the intergroup differences were not significant (Table 2).

The plasma EAA concentrations at midpoint were 664.3 ± 8.8, 606.6 ± 8.5, and 643.3 ± 9.1 μM in the KP, MN and NE groups, respectively. At end point, the plasma EAA concentrations in those groups were 631.3 ± 8.8, 598.6 ± 8.3, and 639.0 ± 8.9 μM, respectively. Plasma EAA concentrations of KP group increased at midpoint compared to baseline, whereas concentrations were unchanged in the NE groups, and decreased in the MN groups. At midpoint and end point, plasma EAA concentrations of KP and NE groups were significantly higher compared to the MN group (Table 2).

The plasma BCAA concentrations at midpoint were 283.7 ± 4.3, 257.2 ± 4.2, and 277.4 ± 4.4 μM in the KP, MN and NE groups, respectively. At end point, the plasma BCAA concentrations in those groups were 284.2 ± 4.3, 264.4 ± 4.1, and 289.1 ± 4.4 μM, respectively. Plasma BCAA concentrations of KP and NE at midpoint and end point increased compared to baseline as MN were decreased or unchanged in the time points compared to baseline. Plasma BCAA concentrations in the KP and NE groups were significantly higher compared to the MN group at both the midpoint and end point (Table 2). Examining the individual BCAAs (valine, leucine and isoleucine) showed a similar trend (Table 2).

### The Correlation Between Delta LAZ and Delta Plasma BCAA Concentration

**Figure 2:**
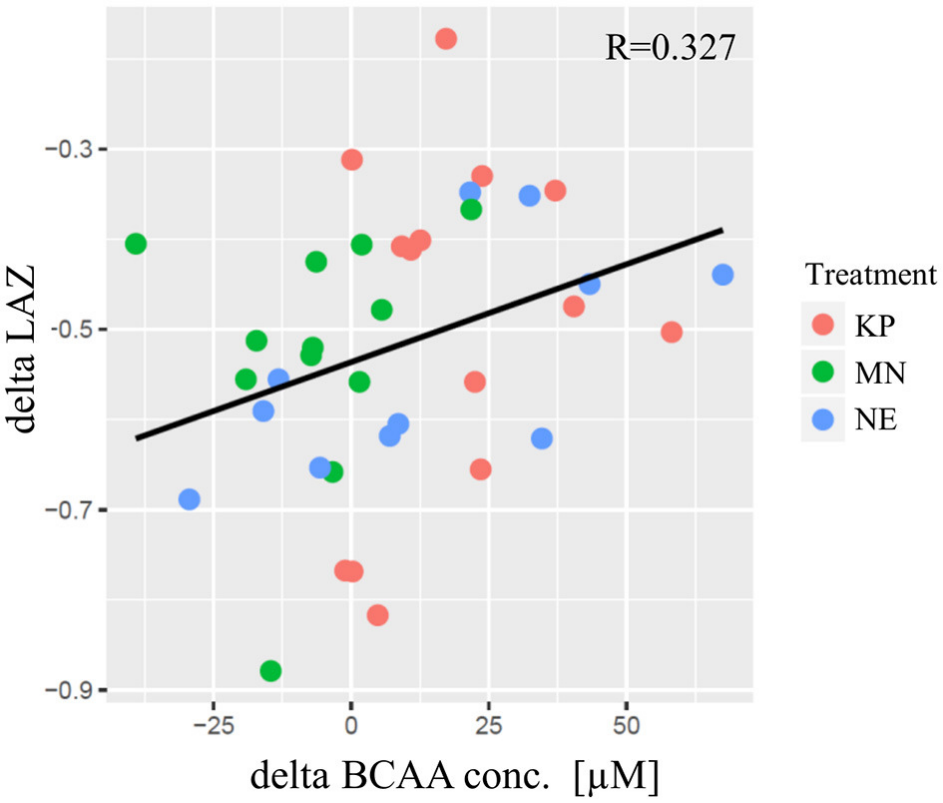
The correlation between delta LAZ and delta plasma BCAA concentration. The horizontal axis indicates the delta of plasma BCAA concentration [μM] from baseline to end point. The vertical axis indicates the delta of LAZ from baseline to end point. Each plot indicate community average. KP, MN, and NE are shown in red, green and blue, respectively. The regression line was shown in black and pearson's correlation was significant (*R* = 0.327; *p* = 0.048). KP, KOKO Plus; MN, Micronutrient; NE, Nutrition Education; BCAA, Branch chain amino acid; LAZ, Length for age z-score.

A significant correlation was found between individual changes in LAZ (delta LAZ) and delta plasma BCAA concentration (*R* = 0.327, *p* = 0.048) (Figure 2).

### Estimated Plasma Amino Acid Concentrations by Number of Supplement Sachets Consumed

At end point, the KP group had estimated plasma BCAA concentrations (± SE) of 270.2 ± 6.4, 284.5 ± 4.2, and 298.8 ± 6.6 μM at consumption levels of 100, 200, and 300 sachets, respectively. In contrast, the estimated plasma BCAA concentrations in the MN group at those consumption levels were 269 ± 6.4, 264.6 ± 4, and 260.3 ± 6.1 μM, respectively (Table 3).

**Table 3:** Estimated plasma BCAA concentration in different number of supplements consumed.

**Supplements consumed**	**Time point**	**Treatment**		***p-*value KP vs. MN**
		**KP**	**MN**	
		**Mean SE**	**Mean SE**	
100	Baseline	264.0 ± 5.2	268.8 ± 5.6	1.000
	Midpoint	274.6 ± 6.5	261.7 ± 6.6	0.500
	End point	270.2 ± 6.4	269.0 ± 6.4	1.000
200	Baseline	265.6 ± 3.9	269.3 ± 3.8	1.000
	Midpoint	283.6 ± 4.2	257.5 ± 4.0	<0.001^***^
	End point	284.5 ± 4.2	264.6 ± 4.0	0.002^**^
300	Baseline	267.3 ± 6.2	269.9 ± 5.9	1.000
	Midpoint	292.7 ± 6.6	253.3 ± 6.1	<0.001^***^
	End point	298.8 ± 6.6	260.3 ± 6.1	<0.001^***^

*Plasma amino acid concentrations were estimated from the following generic model. “Plasma BCAA concentration = ε + β1(treatment) + β2(time point) + β3(supplements consumed) + β4(treatment × time point) + β5(treatment × supplements consumed) + β6(time point × supplements consumed) + β7(treatment × time point × supplements consumed).”*

*P-value of difference between KP group and MN group at the same time point was obtained from general linear hypotheses test (null hypothesis: difference = 0)*.

** and *** *indicate statistically significant high than MN group with p < 0.01 and p < 0.001 at the same time point. KP, KOKO Plus; MN, Micronutrient*.

At end point, the KP group had estimated plasma EAA concentrations of 597.1 ± 13, 632.3 ± 8.34, and 667.6 ± 13.4 μM at consumption levels of 100, 200, and 300 sachets, respectively. In the MN group, on the other hand, the estimated plasma EAA concentrations at those consumption levels were 606.2 ± 12.8, 599 ± 7.8, and 591.8 ± 12.3 μM, respectively (Table 4).

**Table 4:** Estimated plasma EAA concentration in different number of supplements consumed.

**Supplements consumed**	**Time point**	**Treatment**		***p*-value KP vs. MN**
		**KP**	**MN**	
		**Mean SE**	**Mean SE**	
100	Baseline	629.4 ± 10.5	637.9 ± 11.1	1.000
	Midpoint	644.4 ± 13.2	613.9 ± 13.3	0.310
	End point	597.1 ± 13.0	606.2 ± 12.8	1.000
200	Baseline	634.6 ± 7.8	641.4 ± 7.5	1.000
	Midpoint	664.5 ± 8.4	607.1 ± 8.0	<0.001^***^
	End point	632.3 ± 8.3	599.0 ± 7.8	0.011^*^
300	Baseline	639.8 ± 12.7	644.9 ± 12.0	1.000
	Midpoint	684.6 ± 13.4	600.2 ± 12.5	<0.001^***^
	End point	667.6 ± 13.4	591.8 ± 12.3	<0.001^***^

*Plasma amino acid concentrations were estimated from the following generic model. “Plasma EAA concentration = ε + β1(treatment) + β2(time point) + β3(supplements consumed) + β4(treatment × time point) + β5(treatment × supplements consumed) + β6(time point × supplements consumed) + β7(treatment × time point × supplements consumed).”*

*P-value of difference between KP group and MN group at the same time point was obtained from general linear hypotheses test (null hypothesis: difference = 0)*.

* and *** *indicate statistically significant high than MN group with p < 0.05 and p < 0.001 at the same time point. KP, KOKO Plus; MN, Micronutrient*.

The KP group showed dose-dependent increases in plasma BCAA and EAA concentrations with supplement usage, whereas the MN group did not. Plasma BCAA and EAA concentrations were significantly different between the KP and MN groups at both time points for consumption levels of 200 sachets or more (Tables 3, 4).

## Discussion

This secondary analysis embedded in a cluster-randomized single-blind intervention study in Ghana aimed to better understand the effect of a complementary food supplement, KP, on plasma amino acid concentrations. We hypothesized that the complementary macronutrient and micronutrient supplement KP, compared to MN with NE or NE alone, would impact infants' plasma BCAA concentrations. The plasma BCAA concentrations of the KP and NE group significantly increased compared to the MN group at both the midpoint and end point however, there were no differences in the concentrations of KP and NE groups.

KP is designed to meet the amino acid requirements of infants, and lysine was added as an amino acid source to enhance the protein quality of the supplement. The supplement meets 35–55% of amino acid needs based on the total daily requirements of the targeted age group. This is the likely reason why the KP group had a higher predicted lysine concentration than the MN or NE group at midpoint and end point. Intake of KP influences the plasma free lysine concentration of infants.

The predicted plasma EAA and BCAA concentrations of the KP and NE groups were significantly higher than those of the MN group at midpoint and end point. This suggests that the KP group had higher EAA and BCAA concentrations than the MN group, since dietary protein is the key mediator driving the anabolic response of lean tissues, affecting both muscle mass gain and linear bone growth (34). EAAs are of key importance for growing infants and young children because they drive protein synthesis. Leucine, a member of the BCAA family, is not only a precursor but also a key activating factor for protein synthesis *via* the mammalian target of rapamycin signaling pathway (35, 36). An increase in plasma EAA concentration in response to diet is also known to increase protein synthesis in muscle (37). In addition, micronutrients play an important role in protein synthesis, since they are important co-factors for enzymes related to protein synthesis (21, 38). Deficiencies of micronutrients such as zinc are known to limit protein synthesis in animal studies and growth in humans (39, 40). Thus, both amino acids and micronutrients in proper amounts are simultaneously needed to maintain normal homeostasis for protein synthesis and growth. The present study, however, showed no difference in LAZ scores (the primary outcome) between the intervention groups (16). In addition, we cannot explain the EAA and BCAA levels in the NE group. Data on dietary diversity and total energy intake showed that there were no significant differences between the intervention groups observed in this study (16).

The results of this study indicated that, irrespective of the intervention group, clusters of plasma BCAA concentrations were positively correlated with changes in LAZ scores from end point to baseline (Figure 2). This observation was consistent with a report by Semba et al., which showed a strong negative correlation between stunting and circulating EAA levels in a cross-sectional study of children aged 12–59 months in Malawi (6). This result suggests that plasma BCAA levels are related to linear growth. However, associations should not be interpreted as causal relationships; a randomized controlled trial is necessary to adequately test this hypothesis.

Concentrations of plasma amino acids fluctuate with dietary protein intake; therefore, we assessed how supplemental consumption of KP and MN impacted the changes in plasma amino acids. The longitudinal data collection in the study allowed changes in individual infants' plasma amino acid levels to be tracked over time. It was observed that the concentrations of plasma BCAAs were increased at midpoint and end point in the KP group, although not in the MN group, suggesting that intervention with a macronutrient and micronutrient supplement can affect children's plasma amino acid concentrations of children. The study confirms a critical role of BCAAs among plasma amino acids. BCAAs in plasma correlated best with changes in LAZ scores, supporting a key role for BCAAs in linear growth. This marker is the most sensitive variable that correlates with LAZ and might be a promising biomarker for protein nutrition.

The present study has two important limitations: first, the insufficient delivery rates led to low consumption of the supplements; second, the plasma samples for amino acid quantification were collected under suboptimal conditions. The delivery rates of the supplements were insufficient, at <60%, but the consumption rates of the delivered supplements were high, at 84.9% (KP) and 87.2% (MN; further information is provided in the methods section). In real-life scenarios, the most vulnerable population at high risk of stunting is in rural areas with poor infrastructure and limited access to a variety of nutrient dense foods (41). Due to poor delivery of the supplements during intervention, no difference in LAZ could be detected between the groups. After adjusting for compliance, however, we found that LAZ, weight-for-age Z-scores and weight-for-height Z-scores were significantly increased at the end point in the KP group but not the MN group under conditions of optimal delivery and compliance (16). By exploring the dose–response curves of plasma BCAAs and EAAs, it could be predicted that the plasma BCAA and EAA levels of the KP group would significantly increase compared to those of the MN group at midpoint and end point among infants consuming > 200 sachets per year (Tables 3, 4). In the MN group, the estimated EAA concentrations showed a tendency to decrease with time; this tendency became more evident as supplement consumption rose. The results of the present intervention show that KP consumption maintains plasma EAA and BCAA levels, indicating that when sufficient amounts of the supplements are consumed (>200 sachets/year), they may have a beneficial effect on linear growth; the previous report also indicated that the threshold for differences in LAZ between the KP and MN groups was 200 sachets per year (16). However, the generalisability of this finding is limited, and one cannot fully attribute the lack of effect on the plasma amino acid levels to the poor delivery of KP and MN supplements due to inability to compare with the NE intervention group.

Another limitation of this study was the blood sampling conditions, since for ethical reasons, we could not fully control how long the infants fasted before blood collection. Plasma amino acids are known to fluctuate after meals and return to basal levels several hours later. Since the mean fasting interval before the blood draw ranged from 50.2 to 135.9 min at baseline to end point (6 months to 18 months) (Supplementary Figure 1), the plasma amino acid measurements in this study might reflect not only the infants' basal plasma amino acid status but also the nutrients they ingested shortly before blood sampling. However, because there were no differences between sampling time points within the groups, we were able to detect a distinct difference among the groups. The KP and NE groups had higher predicted plasma BCAA and EAA levels than the MN group, and overall BCAA concentrations were correlated with the changes in LAZ. Analysis of our primary outcome has shown a dose–response effect of KP on linear growth (16) as well as BCAA and EAA levels; BCAAs and EAAs increased with consumption in the KP group and decreased with consumption in the MN group.

In conclusion, the concentrations of plasma EAAs and BCAAs in the KP and NE groups were maintained in the infants. Overall plasma BCAA concentrations in infants were correlated with changes in LAZ, irrespective of the treatment group. Adjusting for the consumption of the supplements, we have found that KP may be beneficial for maintaining infants' plasma BCAA concentrations and may support adequate linear growth in children.

## Data Availability Statement

The datasets generated during and/or analyzed during the current study are available from the corresponding author on reasonable request. Requests to access the datasets should be directed to Shibani A. Ghosh, shibani.ghosh@tufts.edu.

## Ethics Statement

The studies involving human participants were reviewed and approved by Ghana Health Service and the Noguchi Memorial Institute for Medical Research at the University of Ghana. Written informed consent to participate in this study was provided by the participants' legal guardian/next of kin.

## Author Contributions

SG, DS, KT-D, and GO designed the research (conception, development of overall research plan, study oversight). CF and HM contributed toward data collection and sample analysis. WS analyzed data and performed statistical analysis. CF and WS wrote the paper. SG had primary responsibility for the final content. All authors reviewed the manuscript.

## Funding

The present study was funded by the Japan International Cooperation Agency (JICA) Preparatory Survey for BOP Business Promotion Project and by Ajinomoto Co., Inc. (Tokyo, Japan).

## Conflict of Interest

CF, WS, and HM are Ajinomoto Co., Inc. employees. DS, GO, KT-D, and SG were supported by grants from both Ajinomoto Co., Inc. (Tokyo, Japan) and Japan International Cooperation Agency (JICA). SG and DS was affiliated at Nevin Scrimshaw International Nutrition Foundation at the time of the intervention trial.

## Publisher's Note

All claims expressed in this article are solely those of the authors and do not necessarily represent those of their affiliated organizations, or those of the publisher, the editors and the reviewers. Any product that may be evaluated in this article, or claim that may be made by its manufacturer, is not guaranteed or endorsed by the publisher.
