# Supplementary material for: Changes of Plasma Amino Acid Profiles in Infants With a Nutrient-Fortified Complementary Food Supplement: Evidence From a 12-Month Single-Blind Cluster-Randomized Controlled Trial
Source: Front Nutr. 2021 Sep 30;8:606002. doi: 10.3389/fnut.2021.606002 (PMC8514778; doi:10.3389/fnut.2021.606002)
Supplement: Supplementary file 3 [file Image_1.pdf]

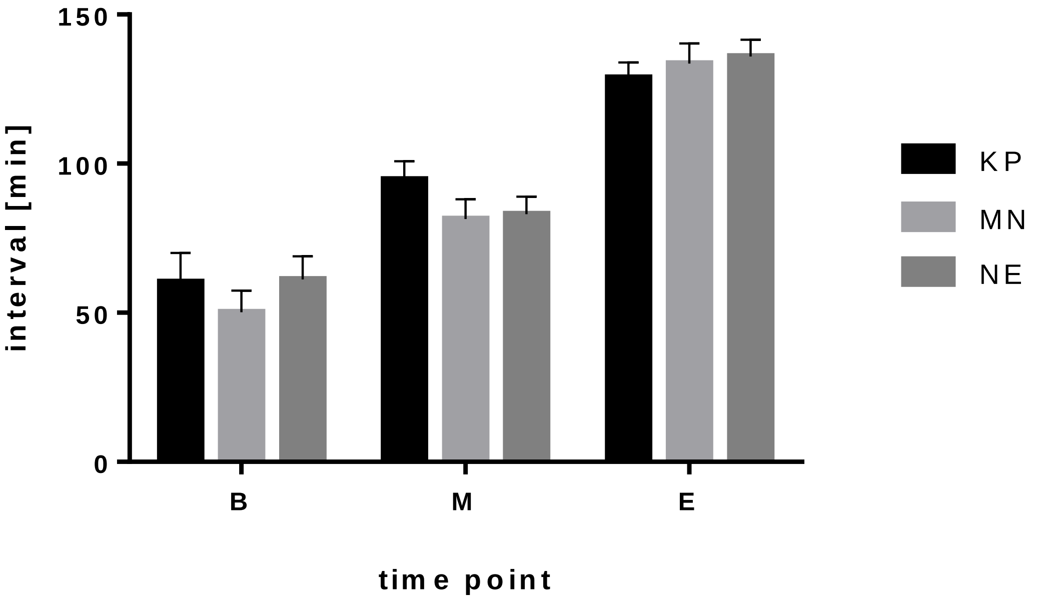

Supplementary figure 1. Fasting interval before blood sampling. The interval was expressed as means ± SEs [μM]. Tukey's multiple comparison was conducted to compare the fasting interval among groups. There are no significant difference between all groups at same time point.

B, Baseline; M, Midpoint; E, End point; KP, KOKO Plus ; MN, Micronutrient ; NE, Nutrition Education.
